# Legal Notes for Institutional Workers

**Published:** 1912-10-19

**Authors:** 


### LEGAL NOTES FOR INSTITUTIONAL WORKERS.

**(The Editor will be pleased to answer Legal queries in this column.)**

### Typhoid Fever an Accident.

Those who are concerned with the Workmen's Compensation Act should bear in mind that persons suffering from certain infectious disorders may sometimes be treated as victims of accident. Some complaints in the nature of "industrial diseases" are specially mentioned in the Act itself, *e.g.*, nystagmus, lead poisoning, etc., but wholly apart from them if it can be proved that infection came about "by accident," the sufferer may obtain compensation. In a case heard some years ago, a man who became infected with anthrax when sorting foreign wool was a successful claimant. In a recent case a bricklayer sought compensation for typhoid fever alleged to have been contracted by him when cleaning out a cesspool. It was proved to the satisfaction of the judge that the cesspool was foul; that, in the course of his work, the man put his pipe in his mouth with his dirty hand, and that the symptoms of typhoid appeared after the lapse of the usual period of incubation from the date of these occurrences. It was also proved that the applicant was a healthy man, and his family with whom he lived were healthy. In these circumstances the judge came to the conclusion that the man was the victim of an accident and that he was entitled to compensation.

### The Law and Medical Ethics.

It cannot be too often impressed upon the practitioner that the rules of professional ethics which he regards as binding upon him are not always binding upon the patient, and are consequently not acted upon in courts of justice. A case recently heard by Judge Howland Roberts at the Clerkenwell County Court is very much in point. The plaintiff, who was one of the medical officers of a Friendly Society, was called in to attend a member of the Society who was suffering from a sore throat. Having seen the patient on Sunday, Monday, and Tuesday, he considered him so much improved that he arranged to come again on Friday. On the Tuesday evening, however, the lad's mother called and said he appeared to be worse. The doctor reassured her, but later on the same day she came to say she had been to see another practitioner who said the boy had quinsy. She asked the plaintiff to come and open it. He refused to do so on the ground that another practitioner had been called in. He now sought to recover from the Society the sum of 17s. 6d. for the visits he had paid. The Society counterclaimed the same sum which had been paid by them for the services rendered by the second practitioner. The plaintiff, in giving his evidence, said that if he was called in to another doctor's patient he refused to see him until he either had that doctor's permission or until the other doctor had been discharged. Where, however, prompt relief was necessary he would treat the patient and inform the relatives that he had seen the patient on behalf of the other doctor and would look to that other doctor for his fee. The second doctor, who attended on subpœna as a witness for the defence, said that when he was called in he did not know that the plaintiff had been consulted. When he ascertained this fact he desired the boy's mother to get the plaintiff to perform the operation, and it was not until after the plaintiff refused that he consented to use his instruments to give the patient relief. In giving evidence he said: "When there is a serious case and no time to write letters to the patient's doctor, I think it is quite sufficient to tell him that the case is urgent. Then the least he can do is to come and see the case. If it is not urgent, he can blow me up for it." The learned judge, after stating that no allegation had been made against the plaintiff's skill, said that the plaintiff had unfortunately omitted to give any weight to the second doctor's opinion. It was not a case where any question of principle arose. It was a question of urgency. The second doctor took all the steps he could take in a case of urgency. When after seeing the boy he was informed that another doctor had been attending him, he advised the mother to go and ask the plaintiff to attend the case, but he refused. The Society had judgment on the claim and counterclaim.

